# Valor Preditivo dos Biomarcadores Cardíacos na Função Tardia do Enxerto em Pacientes com Transplante Renal

**DOI:** 10.36660/abc.20230858

**Published:** 2024-11-07

**Authors:** Rodrigo Pinheiro Amantéa, Virgílio da Rocha Olsen, Laura Caroline Tavares Hastenteufel, Flávia K. Borges, Roberto Ceratti Manfro, Lívia Adams Goldraich, Nadine Clausell

**Affiliations:** 1 Hospital de Clínicas de Porto Alegre Porto Alegre RS Brasil Hospital de Clínicas de Porto Alegre, Porto Alegre, Rio Grande do Sul, RS – Brasil; 2 Santa Casa de Misericórdia de Porto Alegre Porto Alegre RS Brasil Santa Casa de Misericórdia de Porto Alegre, Porto Alegre, RS – Brasil; 3 McMaster University Hamilton Ontario Canadá McMaster University, Hamilton, Ontario – Canadá

**Keywords:** Transplante Renal, Biomarcadores, Assistência Perioperatória

## Introdução

A doença cardiovascular (CV) é a principal causa de morte entre receptores adultos de transplante renal, sendo responsável por 25% das mortes em pacientes com enxertos funcionantes.^1^ Os biomarcadores cardíacos, especialmente o peptídeo natriurético cerebral (BNP) e a troponina cardíaca (cTn), são os biomarcadores mais estudados na predição do risco de eventos cardiovasculares adversos maiores (MACE) na doença renal em estágio terminal e transplante renal.^2,3^

A lesão miocárdica pós cirurgia não cardíaca é uma nova entidade clínica com implicações clínicas e prognósticas relevantes, sendo definida como uma lesão miocárdica isquêmica até 30 dias após a cirurgia não cardíaca, independentemente associada ao aumento da mortalidade.^4^ PJ Devereaux et al. avaliaram a coorte do estudo VISION, que envolveu a avaliação de cTn e BNP perioperatórios em mais de 20.000 pacientes, mas sem um número representativo de pacientes transplantados renais.^5^

Embora muitos centros tenham incluído esses biomarcadores como medidas de rotina em programas de cirurgia não cardíaca, sua aplicabilidade no transplante renal e na predição de desfechos como função tardia do enxerto (FTE) permanece pouco estudada. A FTE é definida como necessidade de hemodiálise na primeira semana pós-operatória e representa um impacto negativo sobre a sobrevivência e a função do enxerto a longo prazo.^6^ Assim, pretendemos avaliar o perfil perioperatório de biomarcadores cardíacos em receptores de transplante renal e explorar sua associação com a FTE e os desfechos CVs pós-operatórios.

## Métodos

### Protocolo de estudo

Conduzimos um estudo de coorte prospectivo, envolvendo pacientes adultos transplantados renais de setembro de 2018 a março de 2020 em um hospital universitário terciário na região sul do Brasil. Todos os pacientes foram submetidos a exames cardíacos antes de entrarem na lista de espera para transplante. Dados clínicos sobre dados demográficos, comorbidades e eventos CV foram coletados. Avaliamos a ocorrência de FTE, que foi definida como a necessidade de diálise na primeira semana após a cirurgia. Os pacientes foram acompanhados durante toda a internação hospitalar e contatados 30 dias após a alta. Os prontuários médicos foram monitorados até um ano após o transplante. A aprovação ética do Comitê de Ética em Pesquisa Institucional foi obtida, bem como o consentimento informado dos pacientes antes da inscrição no estudo.

### Avaliação de biomarcadores

Os níveis de BNP foram avaliados na admissão e 24 horas após o transplante renal usando o ensaio Alere Triage® (MA, EUA). Os níveis de troponina cardíaca de alta sensibilidade (hs-cTn) foram medidos na admissão, 24 horas e 48 horas após o transplante renal. Ambos os ensaios hs-cTnT (Roche®, Basel, Suíça), medidos em n = 81 (75,7%) pacientes, e hs-cTnI (Abbott®, IL, EUA), medidos em n = 26 (24,3%) pacientes, foram usados devido a alterações nas plataformas bioquímicas hospitalares. Para minimizar as diferenças entre as amostras de hs-cTn, analisamos a porcentagem de troponina em relação ao 99º percentil. Um valor de corte de 52 ng/L foi estabelecido, indicando lesão miocárdica, e considerando sua associação prévia com um índice maior de suspeita de isquemia miocárdica^7^ e a faixa de referência laboratorial do nosso hospital para ambos os ensaios. Além disso, um limite mais específico estava representado para nossa população, uma vez que pacientes transplantados renais são conhecidos por apresentarem troponina cardíaca basal elevada. Chesnaye et al. relataram que, em uma coorte de 171 pacientes com doença renal crônica, 170 (99,4%) apresentaram pelo menos uma medição de hs-cTnT acima do 99º percentil da população de referência geral (14 ng/L) em um acompanhamento de 4 anos.^8^ A lesão miocárdica após o transplante foi definida como um aumento maior que 20% na cTn após o transplante em comparação aos níveis pré-operatórios.^9^

### Análise estatística

A análise estatística foi conduzida usando o IBM SPSS Statistics 22 para Windows (IBM Corporation, Somers, NY, EUA). Um valor-p menor que 0,05 foi considerado estatisticamente significativo.

## Resultados

Cento e sete (107) pacientes com transplante renal, com pelo menos uma medição de biomarcadores no perioperatório, foram incluídos prospectivamente. Dados demográficos basais, características clínicas e dados laboratoriais são apresentados na Tabela 1. A FTE ocorreu em 56 pacientes (52,3%). Quatro pacientes apresentaram MACE dentro de 30 dias do transplante (3,7%). Dois pacientes apresentaram infarto do miocárdio sem supradesnivelamento do segmento ST; um paciente morreu após fibrilação ventricular; um paciente apresentou insuficiência cardíaca. Entre os quatro pacientes que apresentaram MACE, dois (50%) apresentaram FTE. O BNP e o Hs-cTn pré-operatórios e pós-operatórios desses pacientes são apresentados na Tabela 2.

**Tabela 1:** – Características basais da coorte do estudo

	n=107
**Idade, anos**	51,4 ± 13,5
**Masculino**	59 (55,1)
**Brancos**	93 (86,9)
**Pretos**	10 (9,3)
**Doença renal primária**	
Desconhecido	34 (31,8)
Nefropatia diabética	14 (13,1)
Nefropatia hipertensiva	11 (10,3)
Outras nefropatias	48 (44,8)
**Comorbidades**	
Diabetes melito	22 (20,6)
Hipertensão	88 (82,2)
Infarto do miocárdio prévio	5 (4,7)
AVC prévio	4 (3,7)
Insuficiência cardíaca	2 (1,9)
Histórico de tabagismo	26 (24,3)
Tempo em diálise, meses	35,5 (20,7-47)
Transplante renal prévio	12 (11,2)
**Índice de risco cardíaco revisado**	2,2 ± 0,5
**Medicamentos**	
Ácido acetilsalicílico	31 (29)
IECA/BRA	37 (34,6)
Betabloqueador	50 (46,7)
Estatina	27 (25,2)
Insulina	15 (14)
**Dados laboratoriais**	
Creatinina (mg/dL)	8,4 ± 3,2
Ureia (mg/dL)	103,2 ± 39,7
Hemoglobina (g/dL)	11,6 ± 2
Glicose (mg/dL)	106 ± 48,4
**Fração de ejeção (%)**	64,6 ± 8,2

Dados expressos como média ± desvio padrão, número absoluto (porcentagem) ou mediana (Q1 - Q3). AVC: acidente vascular cerebral; IECA: inibidor da enzima de conversão da angiotensina; BRA: bloqueador do receptor da angiotensina II.

**Tabela 2:** – Biomarcadores pré-operatórios e pós-operatórios entre pacientes com MACE

Idade, anos	IRCR	Etiologia da DRC	Troponina pré-transplante, ng/L	Troponina pós-transplante, ng/L (24h - 48h)	BNP pré-transplante, pg/mL	BNP pós-transplante, pg/mL (24h)	MACE	Tempo desde o transplante, dias
66	2	Outros	39,64	404,3 - 452,5	345	1230	IAM	0
54	2	Nefropatia por IgA	10	-	-	746	ICC	3
72	3	Nefropatia diabética	14	23 - 434	242	733	IAM	1
80	3	Desconhecido	-	22,7 - 31,8	-	854	PCR/ Morte	2

IAM: infarto agudo do miocárdio; ICC: insuficiência cardíaca congestiva; DRC: doença renal crônica; MACE: eventos cardiovasculares adversos maiores; PCR: parada cardiorrespiratória; IRCR: índice de risco cardíaco revisado.

Foi observada lesão miocárdica pós transplante em 19,6% dos pacientes. Três receptores (2,8%) morreram no primeiro ano pós-transplante. As causas da morte foram sepse, fibrilação ventricular e isquemia mesentérica aguda. Seis pacientes apresentaram nova fibrilação atrial (5,6%), dois tiveram trombose venosa profunda (1,9%) e dois tiveram oclusão arterial aguda (1,9%).

Três pacientes (2,8%) evoluíram com perda do enxerto durante o primeiro ano após o transplante, necessitando de hemodiálise permanente. Todos esses pacientes apresentaram, inicialmente, FTE e necessitaram de enxertectomia. Um deles morreu no 9º dia pós-operatório devido a complicações de isquemia mesentérica. Os demais pacientes com FTE recuperaram a função renal e chegaram ao final do acompanhamento com enxertos funcionantes, sem necessidade de diálise. A creatinina média em 1, 3, 6 e 12 meses após o transplante foi, respectivamente, 1,90 mg/dL ± 0,92, 1,66 mg/dL ± 0,62, 1,65 mg/dL ± 0,79 e 1,51 mg/dL ± 0,71. Pacientes com FTE apresentaram creatinina mais alta em 1 e 3 meses após o transplante (respectivamente p = 0,014 e 0,021). Aos 12 meses, houve uma diferença não estatisticamente significativa entre os dois grupos: 1,38 mg/dL entre os pacientes sem FTE e 1,66 mg/dL entre os pacientes com FTE, p = 0,55.

Um total de 190 pacientes foram submetidos a transplante renal durante o período do estudo, sendo 113 (59,5%) homens e 166 (87,4%) brancos. A nefropatia diabética foi a causa da insuficiência renal em 35 (18,4%) pacientes, e a nefropatia hipertensiva em 26 (13,7%). Um total de 104 pacientes apresentaram FTE (54,7%). A creatinina média aos 12 meses após o transplante foi de 1,53 mg/dL ± 0,68.

### Transplante renal

Os pacientes foram acompanhados pela equipe de transplante renal durante a internação hospitalar. Todos os pacientes foram submetidos a um transplante de doador falecido com morte cerebral, e a imunossupressão consistiu em 500 mg de metilprednisolona intraoperatória seguida de uma dose única de 3 mg/kg de timoglobulina e terapia tripla de manutenção com prednisona, tacrolimus e micofenolato de sódio.

A idade média do doador foi de 45,3 anos ± 17,3; 64 (59,8%) eram brancos e 49 (45,8%) eram do sexo masculino. Trinta e três doadores tinham hipertensão arterial sistêmica (30,8%) e 12 tinham diabetes mellitus (11,2%). O trauma foi a causa da morte de 35 doadores (32,7%) e doença cerebrovascular em 59 doadores (55,1%). O tempo médio de isquemia fria renal foi de 21,45 horas ± 5,22, e a duração média da anastomose cirúrgica do enxerto foi de 27,7 minutos ± 7,7. Avaliamos o índice de perfil de doador renal (KDPI), que representa o tempo pelo qual se espera que um rim de doador falecido funcione em relação aos rins recuperados no ano anterior.^10^ KDPI mais baixos estão associados a uma função estimada mais longa, enquanto KDPI mais altos estão associados a uma função estimada mais curta. O KDPI médio foi de 58% ± 29. Outra variável usada na predição da função renal de doador falecido é o índice de risco de doador renal (KDRI), que representa um escore que estima o risco relativo de falha do enxerto renal pós-transplante, considerando as características do doador falecido.^11^ O KDRI médio foi de 1,38 ± 0,38. A creatinina final média do doador foi de 1,38 mg/dL ± 0,86. Entre essas variáveis, a creatinina final do doador e o KDPI foram associados à FTE na análise univariada. O KDPI médio foi de 52,2% ± 30,2 entre pacientes sem FTE e 63,4% ± 27,6 entre pacientes com FTE, p = 0,048. Além disso, a creatinina final média do doador foi de 1,13 mg/dL ± 0,47 entre pacientes sem FTE e 1,60 mg/dL ± 1,04 entre pacientes com FTE, p = 0,007.

### Peptídeo natriurético cerebral

Os níveis médios de BNP na admissão e 24h após o transplante foram, respectivamente, 234 pg/mL (98,9-611 pg/mL) e 307,5 pg/mL (180-622,5 pg/mL). BNP pré-operatório acima de 100 pg/mL foi observado em 74,6% dos pacientes. Em relação a um corte de valor mediano baseado em 300 pg/mL, a FTE foi associada a níveis de BNP pós-operatórios acima desse valor na regressão logística univariada (OR 2,22, IC de 95%, 1,004-4,908, p = 0,049) e em uma regressão logística multivariada incluindo sexo, idade, diabetes mellitus e IRCR ≥ 3 (OR 2,38, IC de 95%, 1,022-5,520, p = 0,044). Embora o ponto de corte clínico mais consolidado para o BNP seja 100 pg/mL, usado para diagnosticar insuficiência cardíaca, sabe-se que a doença renal reduz a precisão desse teste nessa população, e limites mais altos são necessários para atingir uma melhor estratificação do paciente.^12,13^

### Troponina cardíaca de alta sensibilidade (Hs-cTn)

Os níveis de Hs-cTnI na admissão e em 24 e 48h após o transplante foram respectivamente 12 ng/L (9,7-45,7 ng/L), 10 ng/L (10-27,4 ng/L) e 10,8 ng/L (10-45,9 ng/L). Os níveis de Hs-cTnT na admissão e em 24 e 48h após o transplante foram respectivamente 43,64 ng/L (21,8-79 ng/L), 34,6 ng/L (18,7-54,6 ng/L) e 36 ng/L (16,1-58,3 ng/L). Níveis pré-operatórios de hs-cTn indicativos de lesão miocárdica (>52 ng/L) foram observados em 34,7% dos pacientes, e em 84,7% dos pacientes os níveis excederam o percentil do intervalo de referência 99. Níveis elevados de hs-cTn pré-operatórios também foram preditivos de FTE na regressão logística univariada (OR 5,4, IC de 95%, 1,73-16,85, p = 0,004) e em um modelo multivariado incluindo troponina cardíaca pré-operatória > 52 ng/L, idade, sexo, IRCR ≥ 3 e diabetes mellitus (OR 4,07, IC de 95%, 1,12-14,73, p = 0,032). Em um modelo multivariado, incluindo variáveis relevantes e variáveis de doador estatisticamente significativas na análise univariada (sexo, idade, troponina cardíaca pré-operatória > 52 ng/L, creatinina final do doador e KDPI), tanto a creatinina final do doador quanto a troponina cardíaca pré-operatória > 52 ng/L permaneceram como preditores estatisticamente significativos de FTE (respectivamente OR 6,5, IC de 95%, 1,81-23,09, p = 0,004 e OR 5,16, IC de 95%, 1,12-23,93, p = 0,036).

**Figura 1:**
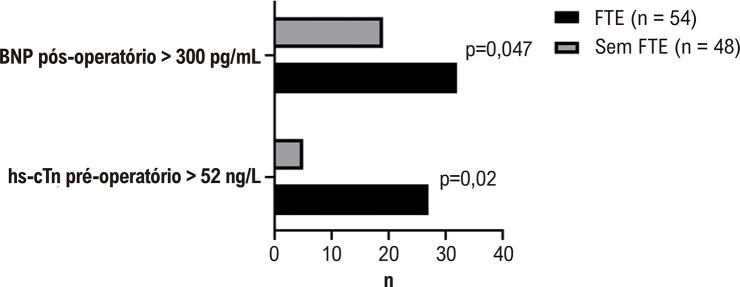
– Biomarcadores cardíacos entre pacientes com e sem função tardia do enxerto. FTE: função tardia do enxerto; BNP: peptídeo natriurético cerebral; hs-cTn: Troponina cardíaca de alta sensibilidade.

A Figura 1 compara a distribuição de pacientes com hs-cTn pré-operatório > 52 ng/L ou BNP pós-operatório > 300 pg/mL entre pacientes com e sem FTE.

## Discussão

Nosso estudo demonstra que a troponina cardíaca basal e o BNP estão elevados em uma grande proporção de receptores de transplante renal, confirmando o perfil de risco CV mais alto para essa população. Além disso, ambos os biomarcadores perioperatórios foram associados à ocorrência de FTE.

Até onde sabemos, este é o primeiro estudo a relatar uma associação de níveis pré-operatórios de hs-cTn com a FTE. Isso pode ser explicado pelo alto risco CV dessa população e seu estado inflamatório crônico predispondo à lesão miocárdica. Os fatores de risco clássicos relacionados à FTE são divididos em aqueles relacionados ao doador (idade do doador, creatinina sérica final e histórico de hipertensão), aqueles relacionados ao receptor (uso de terapia de indução de anticorpos e número de incompatibilidades de HLA ABDR) e aqueles relacionados ao enxerto (especialmente o tempo de isquemia fria).^14^ A troponina cardíaca pré-operatória elevada permaneceu como um preditor estatisticamente significativo de FTE, mesmo quando inserida em um modelo multivariado incluindo variáveis relacionadas ao doador associadas à FTE.

A troponina cardíaca pós-operatória já foi associada a desfechos não cardíacos. Keddis et al. relataram que níveis elevados de troponina na 3ª semana pós-operatória foram associados à ocorrência de FTE no acompanhamento inicial pós-transplante (OR 5,23, IC de 95%, 2,8-9,57, p < 0,0001). Pacientes com FTE também apresentaram valores mais elevados de troponina até 1 ano após a cirurgia (OR 4,37, IC de 95%, 2,53-7,55, p < 0,0001). A persistência de valores elevados deste biomarcador ao longo do acompanhamento neste estudo foi associada à maior ocorrência de desfechos CVs. Por outro lado, pacientes que evoluíram com recuperação progressiva da função renal pós transplante apresentaram maior depuração deste biomarcador e, consequentemente, menores níveis séricos e menor risco de eventos ao longo do acompanhamento.^15^

A FTE já foi previamente associada a doenças relacionadas a um estado inflamatório, como diabetes mellitus e obesidade.^16,17^ Além disso, biomarcadores de inflamação, como TNFalfa, também estavam significativamente elevados entre pacientes que desenvolveram FTE, de acordo com Lauzurica et al.^18^ É importante enfatizar que a FTE permanece como um desfecho perioperatório significativo em transplante renal, uma vez que apresenta consequências clínicas e logísticas/financeiras para o paciente e para o sistema de saúde. Em nossa população, houve uma tendência para diferentes níveis de creatinina até 12 meses após o transplante, sendo a creatinina mais elevada entre pacientes com FTE (significativamente diferente nos primeiros 1 e 3 meses pós-operatórios).

Estudos anteriores indicaram que os níveis de NT-pro-BNP pós transplante podem prever a função do aloenxerto, com níveis mais altos observados em pacientes com FTE.^19^ Wei et al. não observaram alterações significativas na função ventricular esquerda ou na anatomia após o transplante renal, inferindo que a redução progressiva no BNP após o transplante renal foi mais bem explicada pela melhora da função renal e depuração progressiva do peptídeo.^20^ Nesta coorte, observamos uma tendência ascendente nos níveis medianos de BNP após o transplante. Além disso, em concordância com estudos anteriores,^19^ pacientes com FTE apresentaram níveis mais altos de BNP no pós-operatório. Isso pode ser atribuído não apenas à sobrecarga de volume e estresse miocárdico relacionados ao equilíbrio de volume e inflamação, mas também à redução da função renal. O BNP foi apontado como um biomarcador ainda mais sensível e precoce do que a creatinina sérica na predição e diagnóstico de FTE. Um aumento em sua concentração pode ser identificado até 5 dias antes da ocorrência de outras alterações laboratoriais sugestivas de rejeição aguda, como aumento da creatinina sérica, provavelmente devido à retenção subclínica de água.^20^

Nosso estudo apresenta algumas limitações. Primeiramente, trata-se de um estudo de centro único, com um tamanho de amostra limitado. Além disso, houve uma mudança no kit de laboratório hs-cTn durante o estudo. Para minimizar a influência potencial desse problema técnico, analisamos o percentil 99 e a porcentagem de variação da troponina antes e depois do transplante. Outra limitação do nosso estudo é que o limite usado para troponina cardíaca de alta sensibilidade elevada difere dos pontos de corte comumente usados. Essa escolha foi baseada em nosso intervalo de referência do kit de laboratório, que foi igual para hs-cTnI e hs-cTnT, permitindo a maximização das medições de troponina incluídas na análise.

Em conclusão, descobrimos que níveis basais elevados de troponina cardíaca e BNP são comuns nesta população, destacando seu risco CV elevado. As alterações dinâmicas desses marcadores pós-transplante fornecem informações prognósticas valiosas. Nosso estudo contribui para o conhecimento atual como um gerador de hipóteses, sugerindo o potencial do BNP e da troponina na previsão de desfechos não cardíacos, em particular FTE, entre receptores de transplante renal. Esses marcadores representam um recurso adicional na estratificação de risco pré-operatório, auxiliando na criação de estratégias centradas na otimização de desfechos clínicos e permitindo a detecção precoce de pacientes com maior risco de complicações cardíacas e não cardíacas, ambas as quais aumentam a complexidade e os custos envolvidos no gerenciamento de pacientes com transplante renal.
