# Supplementary material for: Valor Preditivo dos Biomarcadores Cardíacos na Função Tardia do Enxerto em Pacientes com Transplante Renal
Source: Arq Bras Cardiol. 2024 Nov 7;121(11):e20230858. [Article in Portuguese] doi: 10.36660/abc.20230858 (PMC11634288; doi:10.36660/abc.20230858)
Supplement: Translation

# Translation: Predictive Value of Cardiac Biomarkers on Delayed Graft Function in Renal Transplant Patients

**DOI:** 10.36660/abc.20230858i

**Authors:** Rodrigo Pinheiro Amantéa, Virgílio da Rocha Olsen, Laura Caroline Tavares Hastenteufel, Flávia K. Borges, Roberto Ceratti Manfro, Lívia Adams Goldraich, Nadine Clausell

**Affiliations:** 1 Brazil Hospital de Clínicas de Porto Alegre, Porto Alegre, Rio Grande do Sul, RS – Brazil; 2 Brazil Santa Casa de Misericórdia de Porto Alegre, Porto Alegre, RS – Brazil; 3 Canada McMaster University, Hamilton, Ontario – Canada

**Keywords:** Kidney Transplantation, Biomarkers, Perioperative Care

## Introduction

Cardiovascular (CV) disease is the leading cause of death among adult kidney transplant recipients, accounting for 25% of deaths in patients with functioning grafts.^1^ Cardiac biomarkers, in particular, brain natriuretic peptide (BNP) and cardiac troponin (cTn), are the most studied biomarkers for predicting the risk of major adverse cardiovascular events (MACE) in end-stage kidney disease and kidney transplant.^2,3^

Myocardial injury after non-cardiac surgery (MINS) is a new clinical entity with relevant clinical and prognostic implications. It is defined as ischemic myocardial injury until 30 days after non-cardiac surgery and is independently associated with increased mortality.^4^ PJ Devereaux et al. evaluated the cohort of the VISION study, which involved the assessment of perioperative cTn and BNP on more than 20.000 patients, but without a representative number of renal transplant patients.^5^

While many centers have included these biomarkers as routine measures in non-cardiac surgery programs, their applicability in kidney transplantation and the prediction of outcomes such as delayed graft function (DGF) remains understudied. DGF is defined as the need for hemodialysis in the first postoperative week and poses a negative impact on survival and long-term graft function.^6^ Therefore, we aim to assess the perioperative profile of cardiac biomarkers in kidney transplant recipients and explore their association with DGF and postoperative CV outcomes.

## Methods

### Study Protocol

We conducted a prospective cohort study involving adult kidney transplant patients from September 2018 to March 2020 at a tertiary academic hospital in southern Brazil. All patients underwent cardiac workup before entering the transplant waiting list. Clinical data on demographics, comorbidities, and CV events were collected. We assessed the occurrence of DGF, which was defined as the need for dialysis within the first week post-surgery. Patients were followed throughout their hospital stay and contacted at 30 days post-discharge. Medical records were monitored until one year after the transplant. Ethical approval from the Institutional Research Ethics Committee was obtained, and informed consent was obtained from patients before study enrollment.

### Biomarker Assessment

BNP levels were evaluated at admission and 24 hours after kidney transplantation using the Alere Triage^®^ assay (MA, USA). High-sensitivity cardiac troponin (hs-cTn) levels were measured at admission, 24 hours, and 48 hours after kidney transplant. Both hs-cTnT (Roche^®^, Basel, Switzerland), measured in n = 81 (75.7%) patients, and hs-cTnI (Abbott^®^, IL, USA) assays, measured in n = 26 (24.3%) patients, were used because of changes in hospital biochemical platforms. To minimize differences between hs-cTn samples, the percentage of troponin relative to the 99^th^ percentile was analyzed. A cutoff value of 52 ng/L was established, indicating myocardial injury, considering its previous association with a higher index of myocardial ischemia suspicion^7^ and our hospital’s laboratory reference range for both assays. It also represented a more specific threshold for our population since renal transplant patients are known to have elevated baseline cardiac troponin. Chesnaye et al. reported that in a cohort of 171 patients with chronic kidney disease, 170 (99.4%) had at least one measurement of hs-cTnT above the 99th percentile of the general reference population (14ng/L) on a 4-year follow-up.^8^ Myocardial injury after transplantation was defined as an increase higher than 20% in cTn after transplant compared to the preoperative levels.^9^

### Statistical analyses

Statistical analysis was conducted using IBM SPSS Statistics 22 for Windows (IBM Corporation, Somers, NY, USA). A p-value of less than 0.05 was considered statistically significant.

## Results

We prospectively included 107 renal transplant patients with at least one measurement of biomarkers perioperatively. Baseline demographics, clinical characteristics, and laboratory data are presented in Table 1. DGF occurred in 56 patients (52.3%). Four patients presented with MACE within 30 days of the transplant (3.7%). Two patients had non-ST-segment elevation myocardial infarction; one patient died after ventricular fibrillation; one patient presented with heart failure. Among the four patients who had MACE, two (50%) had DGF. The preoperative and postoperative BNP and Hs-cTn of these patients are presented in Table 2.

**Table 1:** – Baseline characteristics of the study cohort

	n=107
**Age, years**	51.4 ± 13.5
**Male**	59 (55.1)
**White**	93 (86.9)
**Black**	10 (9.3)
**Primary kidney disease**	
Unknown	34 (31.8)
Diabetic nephropathy	14 (13.1)
Hypertensive nephropathy	11 (10.3)
Other nephropathies	48 (44.8)
**Comorbidities**	
Diabetes mellitus	22 (20.6)
Hypertension	88 (82.2)
Previous myocardial infarction	5 (4.7)
Previous stroke	4 (3.7)
Heart failure	2 (1.9)
History of smoking	26 (24.3)
Time on dialysis, months	35.5 (20.7-47)
Previous kidney transplantation	12 (11.2)
**Revised cardiac risk index**	2.2 ± 0.5
**Medications**	
Acetylsalicylic acid	31 (29)
ACEi/ARB	37 (34.6)
Beta-blocker	50 (46.7)
Statin	27 (25.2)
Insulin	15 (14)
**Laboratory data**	
Creatinine(mg/dL)	8.4 ± 3.2
Urea(mg/dL)	103.2 ± 39.7
Hemoglobin(g/dL)	11.6 ± 2
Glucose(mg/dL)	106 ± 48.4
**Ejection fraction(%)**	64.6 ± 8.2

Data expressed as mean±standard-deviation, absolute number (percentage) or median (Q1 - Q3). ACEi: angiotensin-converting enzyme inhibitor; ARB: angiotensin II receptor blocker.

**Table 2:** – Preoperative and postoperative biomarkers among patients with MACE

Age, years	RCRI	CKD etiology	Pre-transplant troponin, ng/L	Post-transplant troponin, ng/L (24h - 48h)	Pre-transplant BNP, pg/mL	Post-transplant BNP, pg/mL (24h)	MACE	Time from transplant, days
66	2	Others	39.64	404.3 - 452.5	345	1230	AMI	0
54	2	IgA Nephropathy	10	-	-	746	CHF	3
72	3	Diabetic Nephropathy	14	23 - 434	242	733	AMI	1
80	3	Unknown	-	22.7 - 31.8	-	854	CRA / Death	2

AMI: acute myocardial infarction; CHF: congestive heart failure; CKD: chronic kidney disease; CRA: cardiorespiratory arrest; MACE: major adverse cardiovascular events; RCRI: revised cardiac risk index.

Myocardial injury after transplantation was observed in 19.6% of patients. Three recipients (2.8%) died within the first post-transplant year. The causes of death were sepsis, ventricular fibrillation, and acute mesenteric ischemia in one patient each. Six patients presented with new atrial fibrillation (5.6%), two had deep venous thrombosis (1.9%), and two had acute arterial occlusion (1.9%).

Three patients (2.8%) evolved with graft loss during the first year after transplantation, requiring permanent hemodialysis. All of these patients initially presented with DGF and required graftectomy. One of them died on postoperative day 9 due to mesenteric ischemia complications. The remaining patients with DGF recovered renal function and reached the end of follow-up with functioning grafts without the need for dialysis. Mean creatinine at 1, 3, 6 and 12 months after transplantation were respectively 1.90 mg/dL ± 0.92, 1.66 mg/dL ± 0.62, 1.65 mg/dL ± 0.79 and 1.51 mg/dL ± 0.71. Patients with DGF had higher creatinine at 1 and 3 months after transplantation (respectively p = 0.014 and 0.021). At 12 months, there was a non-statistically significant difference between the two groups: 1.38 mg/dL among patients without DGF and 1.66 mg/dL among patients with DGF, p = 0.55.

A total of 190 patients were submitted to renal transplant during the period of the study, 113 (59.5%) male and 166 (87.4%) white. Diabetic nephropathy was the cause of renal failure in 35 (18.4%) patients and hypertensive nephropathy in 26 (13.7%). A total of 104 patients presented DGF (54.7%). The mean creatinine at 12 months after transplantation was 1.53 mg/dL ± 0.68.

### Kidney transplantation

The kidney transplant staff followed patients during their hospital stay. All patients underwent a brain-death deceased donor transplant, and immunosuppression consisted of 500 mg of intra-operative methylprednisolone followed by a single dose of 3 mg/kg of thymoglobulin, and maintenance triple therapy with prednisone, tacrolimus, and sodium mycophenolate.

The mean donor age was 45.3 years ± 17.3; 64 (59.8%) were white, and 49 (45.8%) were male. Thirty-three donors had arterial systemic hypertension (30.8%), and 12 had diabetes mellitus (11.2%). Trauma was the cause of 35 donors’ death (32.7%) and cerebrovascular disease of 59 donors (55.1%). The mean kidney cold ischemia time was 21.45 hours ± 5.22, and the mean graft surgical anastomosis duration was 27.7 minutes ± 7.7. We assessed the kidney donor profile index (KDPI), which synthesizes how long a deceased donor kidney is expected to function relative to the kidneys recovered in the previous year.^10^ Lower KDPI scores are associated with longer estimated functions, while higher KDPI scores are associated with shorter estimated functions. The mean KDPI was 58 % ± 29. Another variable used in the prediction of deceased donor kidney function is the kidney donor risk index (KDRI), which is a score that estimates the relative risk of post-transplant kidney graft failure, considering deceased donor characteristics.^11^ Mean KDRI was 1.38 ± 0.38. The mean final donor creatinine was 1.38 mg/dL ± 0.86. Among these variables, final donor creatinine and KDPI were associated with DGF on univariate analysis. Mean KDPI was 52.2% ± 30.2 among patients without DGF and 63.4% ± 27.6 among patients with DGF, p = 0.048. Also, the mean final donor creatinine was 1.13 mg/dL ± 0.47 among patients without DGF and 1.60 mg/dL ± 1.04 among patients with DGF, p = 0.007.

### Brain natriuretic peptide (BNP)

Median BNP levels at admission and 24h after transplant were respectively 234 pg/mL (98.9-611 pg/mL) and 307.5 pg/mL (180-622.5 pg/mL). Preoperative BNP above 100 pg/mL was observed in 74.6% of patients. Relative to a median value cutoff-based of 300 pg/mL, DGF was associated with postoperative BNP levels above this value on univariate logistic regression (OR 2.22, 95%CI, 1.004-4.908, p = 0.049) and a multivariate logistic regression including sex, age, diabetes mellitus, and RCRI ≥ 3 (OR 2.38, 95% CI, 1.022-5.520, p=0.044). Although the most consolidated clinical cutoff point for BNP is 100 pg/mL, which is used for diagnosing heart failure, kidney disease is known to reduce the accuracy of this test in this population, and higher thresholds are needed to achieve better patient stratification.^12,13^

### High-sensitivity cardiac troponin (Hs-cTn)

Hs-cTnI levels at admission, and at 24 and 48h after transplant were respectively 12 ng/L (9.7-45.7 ng/L), 10 ng/L (10-27.4 ng/L) and 10.8 ng/L (10-45.9 ng/L). Hs-cTnT levels at admission and at 24 and 48h after transplant were respectively 43.64 ng/L (21.8-79 ng/L), 34.6 ng/L (18.7-54.6 ng/L) and 36 ng/L (16.1-58.3 ng/L). Preoperative hs-cTn levels indicative of myocardial injury (>52 ng/L) were observed in 34.7% of patients, and in 84.7% of patients, levels exceeded the 99^th^reference range percentile. Elevated preoperative hs-cTn levels were also predictive of DGF on univariate logistic regression (OR 5.4, 95% CI, 1.73-16.85, p = 0.004) and on a multivariate model including preoperative cardiac troponin > 52 ng/L, age, sex, RCRI ≥ 3 and diabetes mellitus (OR 4.07, 95% CI, 1.12-14.73, p = 0.032). On a multivariate model including relevant variables and those donor variables statistically significant on univariate analysis (sex, age, preoperative cardiac troponin > 52 ng/L, final donor creatinine and KDPI), both final donor creatinine and preoperative cardiac troponin > 52 ng/L remained as statistically significant predictors of DGF (respectively OR 6.5, 95% CI, 1.81-23.09, p = 0.004 and OR 5.16, 95% CI, 1.12-23.93, p = 0.036).

Figure 1 compares the distribution of patients with preoperative hs-cTn> 52 ng/L or postoperative BNP>300 pg/mL between patients with and without DGF.

**Figure 1:** – Cardiac biomarkers among patientes with and without delayed graft function. hs-cTn: High-sensitivity cardiac troponin; BNP: Brain natriuretic peptide; DGF: delayed graft function.

## Discussion

Our study demonstrates that baseline cardiac troponin and BNP are elevated in a large proportion of kidney transplant recipients, confirming the higher CV risk profile for this population. Additionally, both perioperative biomarkers were associated with the occurrence of DGF.

To the best of our knowledge, this is the first study to report an association of preoperative levels of hs-cTn with DGF. This may be explained by the high CV risk of this population and their chronic inflammatory status predisposing to myocardial injury. Classic risk factors related to DGF are divided into donor-related (donor’s age, final serum creatinine and history of hypertension), recipient-related (use of antibody induction therapy and number of HLA ABDR mismatches) and graft-related (particularly cold ischemia time).^14^ Elevated preoperative cardiac troponin remained a statistically significant predictor of DGF even when inserted into a multivariate model including donor-related variables associated with DGF.

Postoperative cardiac troponin has already been associated with non-cardiac outcomes. Keddis et al. reported that elevated troponin levels on the 3rd postoperative week were associated with the occurrence of DGF in the initial post-transplant follow-up (OR 5.23, 95% CI, 2.8-9.57, p < 0.0001). Patients who presented DGF also had higher troponin values up to 1 year after surgery (OR 4.37, 95% CI, 2.53-7.55, p < 0.0001). The persistence of high values of this biomarker throughout follow-up in this study was associated with a greater occurrence of CV outcomes. Conversely, patients who evolved with the progressive recovery of renal function after transplantation achieved greater clearance of this biomarker, and consequently lower serum levels and lower risk of events throughout follow-up.^15^

DGF has been previously associated with diseases related to an inflammatory state, such as diabetes mellitus and obesity.^16,17^ Furthermore, biomarkers of inflammation, such as TNFalfa, were also significantly elevated among patients who developed DGF according to Lauzurica et al.^18^ It is important to emphasize that DGF remains a significant perioperative outcome in renal transplant, as it poses both clinical and logistic/financial consequences to the patient and to the healthcare system. In our population, there was a trend toward different creatinine levels up to 12 months after transplantation, being creatinine more elevated among patients with DGF (significantly different in the first 1 and 3 postoperative months).

Previous studies have indicated that NT-pro-BNP levels after transplant may predict allograft function, with higher levels observed in patients with DGF.^19^ Wei et al. observed that no significant changes were seen in left ventricular function or anatomy after renal transplantation, inferring that the progressive reduction in BNP after renal transplantation was better explained by renal function improvement and progressive depuration of the peptide.^20^ In this cohort, we observed an ascending trend in median BNP levels after transplant. Moreover, in agreement with previous studies,^19^ patients with DGF exhibited higher postoperative BNP levels. This may be attributed not only to volume overload and myocardial stress related to volume balance and inflammation but also to reduced renal functioning. BNP has been pointed out as an even more sensitive and early biomarker than serum creatinine in the prediction and diagnosis of DGF. An increase in its concentration can be identified up to 5 days before the occurrence of other laboratory changes suggestive of acute rejection, such as serum creatinine increase, probably due to subclinical water retention.^20^

Our study has some limitations. First, it is a single-center study with a limited sample size. Additionally, there was a change in the hs-cTn laboratory kit during the study. To minimize the potential influence of this technical issue, we analyzed the 99^th^ percentile and the percentage of troponin variation before and after transplant. Another limitation of our study is that the threshold used for elevated high-sensitivity cardiac troponin differs from the commonly used cutoffs. Its choice was based on our laboratory kit reference range, which was equal for hs-cTnI and hs-cTnT, allowing the maximization of troponin measurements included in the analysis.

In conclusion, we found that elevated baseline levels of cardiac troponin and BNP are common in this population, highlighting their heightened CV risk. The dynamic changes in these biomarkers post-transplant provide valuable prognostic information. Our study adds to the current knowledge as a hypothesis generator, suggesting BNP and troponin’s potential in predicting non-cardiac outcomes, in particular DGF, among renal transplant recipients. They may represent an additional resource on preoperative risk stratification, aiding in the creation of strategies focused on optimizing clinical outcomes and enabling early detection of patients at higher risk of cardiac and non-cardiac complications, both of which increase the complexity and costs involved in renal transplant patient management.
